# Action of Vitamin D and the Receptor, VDRa, in Calcium Handling in Zebrafish (*Danio rerio*)

**DOI:** 10.1371/journal.pone.0045650

**Published:** 2012-09-19

**Authors:** Chia-Hao Lin, Che-Hsien Su, Deng-Yu Tseng, Feng-Chun Ding, Pung-Pung Hwang

**Affiliations:** 1 Institute of Cellular and Organismic Biology, Academia Sinica, Taipei, Taiwan, ROC; 2 Institute of Fishery Science, National Taiwan University, Taipei, Taiwan, ROC; 3 Department of Biological Sciences and Technology, National University of Tainan, Tainan, ROC; University of Oldenburg, Germany

## Abstract

The purpose of the present study was to use zebrafish as a model to investigate how vitamin D and its receptors interact to control Ca^2+^ uptake function. Low-Ca^2+^ fresh water stimulated Ca^2+^ influx and expressions of *epithelial calcium channel* (*ecac*), *vitamin D-25-hydroxylase* (*cyp2r1*), *vitamin D receptor a* (*vdra*), and *vdrb* in zebrafish. Exogenous vitamin D increased Ca^2+^ influx and expressions of *ecac* and *25-hydroxyvitamin D_3_-24-hydroxylase (cyp24a1)*, but downregulated *1α-OHase* (*cyp27b1*) with no effects on other Ca^2+^ transporters. Morpholino oligonucleotide knockdown of VDRa, but not VDRb, was found as a consequence of calcium uptake inhibition by knockdown of *ecac*, and ossification of vertebrae is impaired. Taken together, vitamin D-VDRa signaling may stimulate Ca^2+^ uptake by upregulating ECaC in zebrafish, thereby clarifying the Ca^2+^-handling function of only a VDR in teleosts. Zebrafish may be useful as a model to explore the function of vitamin D-VDR signaling in Ca^2+^ homeostasis and the related physiological processes in vertebrates.

## Introduction

In vertebrates, one of physiological roles of Ca^2+^ is its involvement in bone formation. Decrease of renal Ca^2+^ reabsorption and intestinal Ca^2+^ absorption is an important factor causing osteoporosis [1]. Vanoevelen et al (2011) provided both genetic and functional evidence that transcellular epithelial Ca^2+^ uptake is vital to sustain life and enable bone formation [2]. Therefore, regulating Ca^2+^ uptake is highly essential to vertebrate life. The major source of Ca^2+^ in terrestrial vertebrates is from food. Fish, unlike terrestrial vertebrates, continually face ambient water with variable Ca^2+^ levels and absorb Ca^2+^ from the surrounding water. In adult fish, the predominant route of Ca^2+^ entry from the environment is across the gill epithelium, while in larvae, the body skin is the major route of Ca^2+^ uptake before full development of the gills occurs [3], [4]. Both terrestrial and aquatic vertebrates share similar mechanisms of Ca^2+^ uptake in specific cells and organs. According to the current model in mammals and teleosts, active transcellular Ca^2+^ transport is carried out through the operation of apical epithelial Ca^2+^ channels (ECaC), and the basolateral plasma membrane Ca^2+^-ATPase (PMCA) and Na^+^/Ca^2+^ exchanger (NCX) [5]–[8].

Vitamin D is well documented as vital endocrine regulating Ca^2+^ uptake in mammals. A vitamin D precursor is initially synthesized in the skin. Through a series of reactions, vitamin D-25 hydroxylase (CYP2R1) converts the vitamin-D precursor into 25-hydroxyvitamin D_3_ (25(OH)D_3_), which is then converted to 1α,25-dihydroxytamin D_3_ (1α,25(OH)_2_D_3_), the active form of vitamin D, by renal 1α-OHase (CYP27B1) [9]. The 1α,25(OH)_2_D_3_ level is modulated by 25-hydroxyvitamin D_3_-24-hydroxylase (CYP24A1). CYP24A1, a mitochondrial enzyme in target cells, functions to degrade 1α,25(OH)_2_D_3_ [10]. Both the endocrine synthesis of 1α,25(OH)_2_D_3_ in the kidneys and degradation of this hormone at peripheral sites are associated with the homeostasis of 1α,25(OH)_2_D_3_ in mammals. Although 1α,25(OH)_2_D_3_ was also detected in the lamprey, one of the earliest vertebrate lacking a calcified skeleton and teeth, it was found to play a non-calcemic role there [11]. Those results imply that vitamin D_3_ may initially have evolved a Ca^2+^ regulatory function in bony vertebrates. From evolutionary and physiological points of view, teleosts have been an important model to explore the hypothesis of whether vitamin D also has a calcemic function in bony vertebrates because Ca^2+^ uptake mechanisms of teleosts were demonstrated to be similar to those of mammals as described above. In teleosts 1α,25(OH)_2_D_3_ was also demonstrated to be produced by renal tissues and the liver [12]–[14], and CYP24A1, CYP2R1, and CYP27B1 were also identified in teleosts [15]–[17]. Vitamin D was reported to elevate the serum Ca^2+^ level in carp and cod [18], [19]. Sea bream with a vitamin D-deficient diet showed reduced growth and lower Ca^2+^ turnover [20]. Changes in 1α,25(OH)_2_D_3_ concentrations and expressions of vitamin D receptor (VDR) were noted in Atlantic salmon undergoing smoltification and migrating from fresh water (with low Ca^2+^ concentrations) to seawater (with high Ca^2+^ concentrations), suggesting that regulation of the synthesis of 1α,25(OH)_2_D_3_ and VDR is dependent upon ambient Ca^2+^ concentrations [21]. However, detailed mechanisms of how vitamin D regulates the Ca^2+^ uptake function in teleosts are still largely unclear.

The vitamin D receptor (VDR), a ligand-activated transcription factor, forms a vitamin D_3_-VDR complex upon binding with vitamin D. This complex could upregulate mammalian intestinal *ecac* translation by binding the vitamin D_3_-responsive element (VDRE) in the promoter region of *ecac* [22], and consequently enhances the function of Ca^2+^ absorption, which is an important pathway for controlling Ca^2+^ homeostasis in mammals [9]. Recently in an ecac-defective zebrafish mutant, Vanoevelen et al. [2] demonstrated the importance of ECaC in bone formation. In the ecac promoter region of zebrafish and fugu (*Takifugu rubripes*), putative VDREs were also identified by a bioinformatics analysis [17], [23]. However, there is still no molecular physiological evidence to clarify target cells (ionocytes) or transporters (ECaC, NCX, and PMCA) that are regulated by vitamin D-VDR signaling pathway in teleosts. Teleosts have 2 paralogous VDR because of whole-genome duplication [24], [25]. Although most gene-duplication events are non-functional and eventually result in gene loss, about 20%∼50% of paralogous genes are conserved as one of the duplicates acquires a new function or subfunction [26]. Exploring if 2 paralogous VDRs have different roles in calcemic regulatory functions in teleosts has been a challenging issue. Mammalian cell lines expressing teleost VDRs were found to induce transcription of VDRE-containing expression constructs with 1α,25(OH)_2_D_3_ [24], [27], [28]. On the other hand, 2 isoforms of medaka VDR showed different responses to 1α,25(OH)_2_D_3_ and induced different transcripts of VDRE-containing expression constructs in cell lines [24]. These studies in vitro suggested that VDRs are activated by 1α,25(OH)_2_D_3_, and the 2 paralogous VDRs may undergo functional divergence in teleosts; however, these notions lack in vivo molecular/physiological evidence to support them.

Zebrafish with a well-established genomic database and advantages of morpholino gene knockdown technique is a competent model for research on ion regulation and related endocrine controls [3], [17], [29]–[31]. In zebrafish gills and, in embryonic stages, the skin, a specific ionocyte type that expresses ECaC, PMCA2, and NCX1b was identified to be responsible for transepithelial Ca^2+^ uptake function [3], [4], [8], [32], [33], and this provides an excellent platform to further explore vitamin D's control of Ca^2+^ uptake mechanisms. The purpose of the present study was to use zebrafish to clarify the molecular physiological mechanisms of vitamin D control of epithelial Ca^2+^ transport in teleosts. Experiments were designed to address 2 specific questions: (1) does the vitamin D system control zebrafish Ca^2+^ uptake function by regulating the Ca^2+^ transporters, *ecac*, *ncx1b*, and/or *pmca2*? and (2) does vitamin D control zebrafish Ca^2+^ uptake function through 1 or both of the paralogous VDRs, VDRa and VDRb? Hence, the effects of environmental Ca^2+^ levels and exogenous vitamin D on Ca^2+^ contents and influx, and the mRNA expressions of Ca^2+^ transporters (*ecac, ncx1b,* and *pmca2*) and vitamin D-related genes (*vdra*, *vdrb*, *cyp24a1*, *cyp2r1,* and *cyp27b1*) were investigated. Moreover, the effects of knockdown of VDRa or VDRb on Ca^2+^ contents/influx and the expression of Ca^2+^ transporters and Ca^2+^-regulatory endocrines in zebrafish embryos were also examined.

## Methods

### Experimental Animals

The wild-type AB strain of zebrafish (*Danio rerio*) were kept in local tap water ([Ca^2+^] of 0.2 mM) at 28.5°C under a 14∶10-h light-dark photoperiod at the Institute of Cellular and Organismic Biology, Academia Sinica, Taipei, Taiwan. Experimental protocols were approved by the Academia Sinica Institutional Animal Care and Utilization Committee (approval no.: RFIZOOHP220782).

### Acclimation Experiments

Artificial fresh water with high- (2 mM) or low-Ca^2+^ (0.02 mM) levels was prepared with double-deionized water (model Milli-RO60; Millipore, Billerica, MA, USA) supplemented with adequate CaSO_4_·2H_2_O, MgSO_4_·7H_2_O, NaCl, K_2_HPO_4_, and KH_2_PO_4_. Ca^2+^ concentrations (total calcium levels measured by absorption spectrophotometry) of high- and low-Ca^2+^ media were 2 and 0.02 mM, respectively, but the other ion concentrations of the 3 media were the same ([Na^+^], 0.5 mM; [Mg^2+^], 0.16 mM; and [K^+^], 0.3 mM) as those in local tap water. Variations in ion concentrations were maintained within 10% of the predicted values. Fertilized zebrafish eggs were transferred to high- and low- Ca^2+^ media, respectively, and incubated thereafter until sampling at 3 or 5 d post-fertilization (dpf).

### Vitamin D (1α,25(OH)_2_D_3_ ) Incubation Experiments

1α,25(OH)_2_D_3_ (cat. no. 32222-06-3, Sigma, St. Louis, MO, USA) was dissolved in 95% ethanol at 0 (control) and 20 µg/l (0.48 nM). Zebrafish embryos were incubated in 1α,25(OH)_2_D_3_-containing media immediately after fertilization, and were sampled at 3 dpf for subsequent analysis. Incubation media were changed with a new 1α,25(OH)_2_D_3_ solution every day to maintain constant levels of 1α,25(OH)_2_D_3_. During incubation, neither significant mortality nor abnormal behavior was found.

### Whole-body Ca^2+^ Content

Three-dpf zebrafish embryos were anesthetized with 0.2% buffered MS-222 (Sigma) and then briefly rinsed in deionized water. Thirty individuals were pooled as 1 sample. HNO_3_ (13.1 N) was added to samples for digestion at 60°C overnight. Digested solutions were diluted with double-deionized water, and the total calcium content was measured with a Z-8000 atomic absorption spectrophotometer (Hitachi, Tokyo, Japan). Standard solutions (Merck, Darmstadt, Germany) were used to make the standard curves.

### Whole-body Ca^2+^ Influx

By following previously described methods [34] with some modifications, zebrafish embryos were dechorionated, rinsed briefly in deionized water, and then transferred to 2 ml of ^45^Ca^2+^ (Amersham, Piscataway, NJ, USA; with a final working specific activity of 1∼2 mCi/mmol)-containing medium for a subsequent 4-h incubation. After incubation, embryos were washed 4 times in fresh water without isotope. Six embryos were pooled into 1 vial, anesthetized with 0.2% buffered MS-222, and digested with tissue solubilizer (Solvable; Packard, Meriden, CT, USA) at 60°C for 8 h. The digested solutions were supplemented with counting solution (Ultima Gold; Packard), and the radioactivities of the solutions were counted with a liquid scintillation beta counter (LS6500; Beckman, Fullerton, CA, USA). The Ca^2+^ influx was calculated using the following formula: *J*_in_ = Q_embryo_ X_out_^−1^ *t* ^−1^; where *J*_in_ is the influx (pmol·h ^−1^), Q_embryo_ is the radioactivity of the embryo (cpm per individual) at the end of incubation, X_out_ is the specific activity of the incubation medium (cpm/pmol), *t* is the incubation time (h), The influx was expressed in pmol·mg^−1^·h^−1^ by dividing *J*_in_ by the embryo wet weight (mg). Both the data of Q_embryo_ and W were from pooled samples, and the averaged values were used during calculation.

### RNA Extraction

After anesthetization with 0.03% MS222, appropriate amounts of zebrafish tissues or embryos were collected and homogenized in 1 ml Trizol reagent (Invitrogen, Carlsbad, CA, USA), then mixed with 0.2 ml chloroform, and thoroughly shaken. After centrifugation at 4°C and 12,000×*g* for 30 min, supernatants were obtained. Samples were mixed with an equal volume of isopropanol. Pellets were precipitated by centrifugation at 4°C and 12,000×*g* for 30 min, washed with 70% alcohol, and stored at −20°C until use.

### Reverse-transcription Polymerase Chain Reaction (RT-PCR) Analysis

For complementary (c)DNA synthesis, 1∼5 µg of total RNA was reverse-transcribed in a final volume 20 µl containing 0.5 mM dNTPs, 2.5 µM oligo (dT)_20_, 250 ng random primers, 5 mM dithiothreitol, 40 units RNase inhibitor, and 200 units Superscript RT (Invitrogen) for 1 h at 50°C followed a 70°C incubation for 15 min. For PCR amplification, 2 µl cDNA was used as a template in a 50-µL final reaction volume containing 0.25 mM dNTPs, 2.5 units Taq polymerase (Takara, Shiga, Japan), and 0.2 µM of each primer (Table S1). Thirty cycles were performed for each reaction. All amplicons were sequenced to ensure that the PCR products were the desired gene fragments.

### Quantitative Real-time (q)PCR

A qPCR was performed with a Light Cycler real-time PCR system (Roche, Penzberg, Germany) in a final volume of 10 µl, containing 5 µl 2x SYBR Green I Master Mix (Roche), 300 nM of the primer pairs, and 20∼30 ng cDNA. The standard curve for each gene was checked in a linear range with β-actin as an internal control. The primer sets for the qPCR are shown in Table S1.

### Bioinformatic Analysis

All protein sequences were obtained from the Ensembl and NCBI databases. The accession number of the sequence were as follows: zebrafish (*Danio rerio*) VDRa (NP_570994), zebrafish VDRb (NP_001153457), flounder (*Paralichthys olivaceus*) VDRa (BAA95016), flounder VDRb (BAA95015), frog (*Xenopus laevis*) VDR (NP_001079288), chicken (*Gallus gallus*) VDR (NP_990429), human (*Homo sapiens*) VDR (BAH02291), medaka (*Oryzias latipes*) VDRα (NP_001121988), medaka VDRβ (NP_001121989), Nile tilapia (*Oreochromis niloticus*) VDRa (XP_003449167), Nile tilapia VDRb (XP_003441588), Atlantic salmon VDR (NP_001117029), stickleback (*Gasterosteus aculeatus*) VDRa (ENSGACT00000006308), stickleback VDRb (ENSGACT00000010601), seabass (*Dicentrarchus labrax*) VDRb (CBN80914), mouse (*Mus musculus*) VDR (NP_033530), lizard (*Anolis carolinensis*) VDR (ENSACAT00000013576), and lamprey (*Petromyzon marinus*) VDR (AAP05810). Alignment of the amino-acid sequences was conducted using ClustalW via the SDSC Biology Workbench (http://workbench.sdsc.edu). Phylogenetic analyses were carried out using the Neighbor-joining method. Six hundred bootstrap replicate analyses were carried out with MEGA5.0 software.

### In-situ Hybridization

Zebrafish *ecac* (NM_001001849, full length of the open reading frame) or *vdra* (NW_003336067.1, nt114962∼115564) fragments were obtained by a PCR and inserted into the pGEM-T easy vector (Promega, Madison, WI, USA). The inserted fragments were amplified with the T7 and SP6 primers by a PCR, and the products were used as templates for in vitro transcription with T7 and SP6 RNA polymerase (Roche) in the presence of digoxigenin (DIG)-UTP (Roche) to synthesize sense and antisense probes, respectively. Zebrafish embryos were anesthetized on ice and fixed with 4% paraformaldehyde (PFA) in a phosphate-buffered saline solution (PBS; 1.4 mM NaCl, 0.2 mM KCl, 0.1 mM Na_2_HPO_4_, and 0.002 mM KH_2_PO_4_; pH 7.4) at 4°C overnight. To perform the in situ hybridization, we followed a previous description [32]. For quantitative analysis, the numbers of *ecac*-expressing cells in 12 randomly-selected areas (100×100 µm each) on the yolk-sac surface of an embryo were counted.

### Morpholino Oligonucleotide (MO) Knockdown

The zebrafish VDRa MO (5′- AACGGCACTATTTTCCGTAAGCATC-3′) and VDRb MO (5′- AACGTTCCGGTCGAACTCATCTGGC-3′) were prepared with 1x Danieau solution (58 mM NaCl, 0.7 mM KCl, 0.4 mM MgSO_4_, 0.6 mM Ca(NO_3_)_2_, and 5.0 mM HEPES; pH 7.6). A standard control MO (5′-CCTCTTACCTCAGTTACAATTTATA-3′) was used as the control. To analyze the physiological function of VDRa/b under normal development, we chose to inject 4 ng/embryo of the MO into embryos in the following experiments. At this dose, neither significant mortality nor abnormal behavior was found. The MOs (4 ng/embryo) were injected into embryos at the 1∼2-cell stage using an IM-300 microinjector system (Narishige Scientific Instrument Laboratory, Tokyo, Japan). MO-injected embryos at 3 dpf were sampled for subsequent analyses.

### Western Blot Analysis

Thirty embryos were pooled as 1 sample and homogenized. Protein at 50 µg/well was loaded onto 10% sodium dodecylsulfate polyacrylamide gel electrophoresis (SDS-PAGE) at 100 V for 2 h. After separation, proteins were transferred onto a polyvinylidene difluoride membrane (Millipore, Billerica, MA, USA) at 100 V for 2 h. After being blocked for 1.5 h in blocking buffer, blots were incubated with zebrafish VDRa or VDRb polyclonal antibodies overnight at 4°C, diluted 1∶1000 with alkaline-phosphatase-conjugated goat anti-rabbit immunoglobulin G (IgG) (diluted 1∶2500, at room temperature; Jackson Laboratories, USA) for another 2 h. Blots were developed with 5-bromo-4-chloro-3-indolylphosphate/nitro-blue tetrazolium.

### Vertebrae Staining

Zebrafish embryos were incubated in the staining solution (0.2% calcein, Sigma) for 10 min. After incubation, embryos were washed with fresh water, and then euthanized in MS-222. Observations were carried out using a microscope with a green-fluorescence filter set.

### Cryosectioning

Fresh zebrafish gills were fixed with 4% PFA at 4°C for 3 h and then immersed in PBS containing 5%, 10%, and 20% sucrose for 15 min each at room temperature. Finally, gills were soaked in a mixed PBS solution (OCT compound: 20% sucrose at 1∶2) overnight and embedded with OCT compound embedding medium (Sakura, Tokyo, Japan) at 20°C. Cryosections at 6 µm were made with a cryostat (CM 1900; Leica, Heidelberg, Germany), and these were placed onto poly-L-lysine-coated slides (EMS, Hatfield, PA, USA).

### Immunocytochemistry

Prepared slides were rinsed in PBS and blocked with 3% bovine serum albumin (BSA) for 30 min. Afterward, slides were first incubated with an α5 monoclonal antibody against the α-subunit of avian Na,K-ATPase (NKA) (Hybridoma Bank, University of Iowa, Ames, IA, USA; 1∶600 dilution) overnight at 4°C. Slides were washed twice with PBS and incubated with an Alexa Fluor 568 goat anti-mouse IgG antibody (Molecular Probes, Carlsbad, CA, USA; 1∶200 diluted with PBS) for 2 h at room temperature. Images were acquired with a Leica TCS-NT confocal laser scanning microscope (Leica) or an Axioplan 2 imaging microscope.

### Statistical Analysis

Group data sets were confirmed to be normal distribution by Anderson Darling Normality Test (*p*>0.05).Data are presented as the mean ± SD and were analyzed by one-way analysis of variance (ANOVA) and Student’s *t*-test.

## Results

### Effects of Environmental Ca^2+^ Levels on Messenger (m)RNA Expressions of Ca^2+^-Related Genes

**Figure 1:**
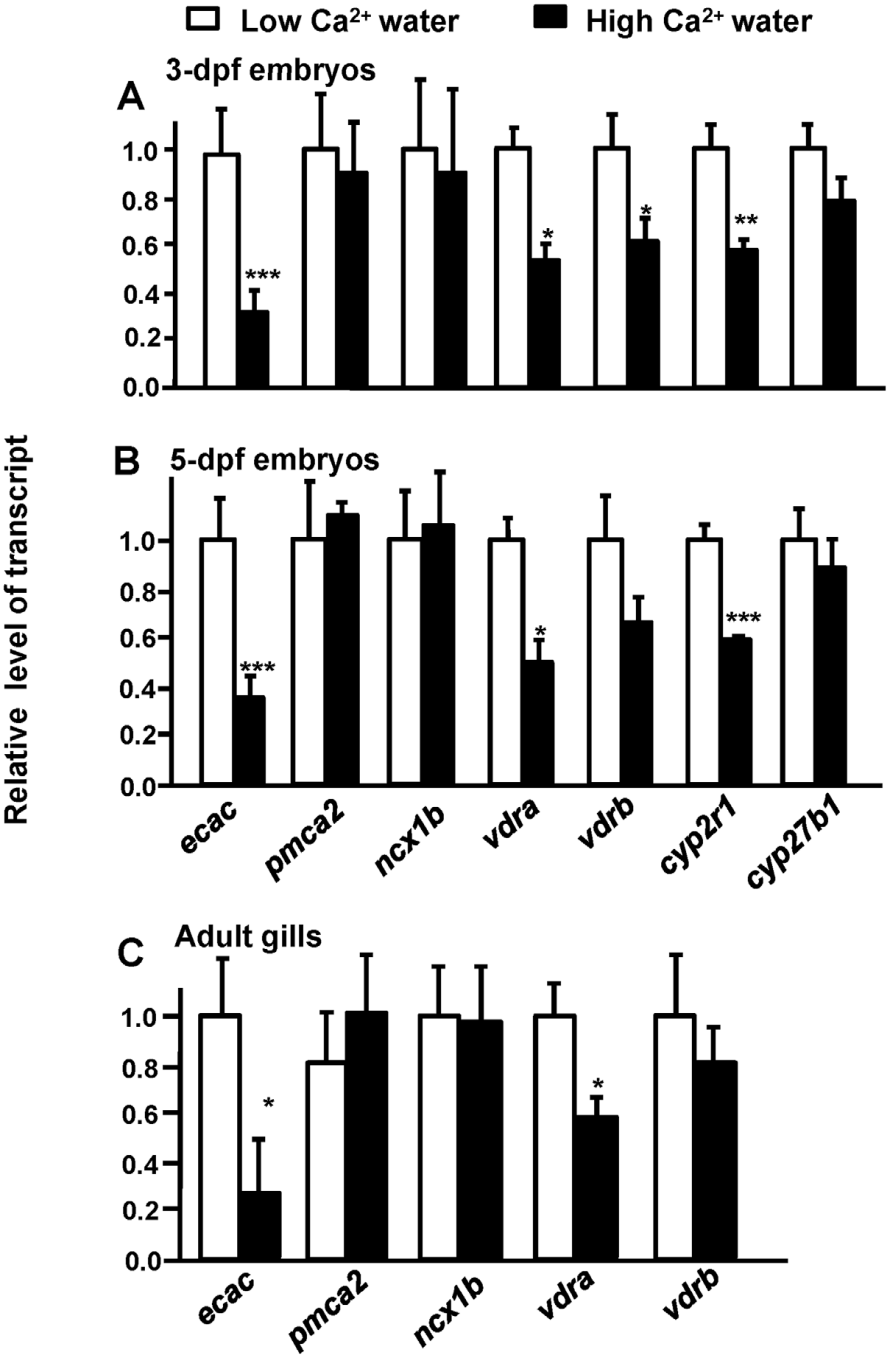
Effect of different Ca^2+^ concentrations on mRNA expressions of Ca^2+^-related genes. (A) mRNA expressions in 3-d post-fertilization (dpf) zebrafish embryos. (B) mRNA expressions in 5-dpf zebrafish embryos. (C) mRNA expressions in gills of adult zebrafish. mRNA expressions were analyzed by a qPCR, and values were normalized to β-actin. Values are the mean ± SD (*n* = 4∼6). Student’s *t-test*, *p<0.05; **p<0.05; ***p<0.001.

After acclimation for 3 or 5 d in artificial fresh water containing different levels of Ca^2+^, zebrafish *ecac*, *vdra,* and *cyp2r1* mRNA expressions were significantly stimulated by low-Ca^2+^ water. On the contrary, *pmca2*, *ncx1b,* and *cyp27b1* mRNA expressions were not affected by environmental Ca^2+^ levels. Environmental Ca^2+^ levels produced different effects on *vdrb* expression at 3 and 5 dpf. Low-Ca^2+^ water stimulated *vdrb* expression in 3-dpf embryo, but *vdrb* expression was not affected at 5 dpf (Fig. 1A, B). Furthermore, mRNA expressions of *ecac*, *pmca2*, *ncx1b, vdra*, and *vdrb* were also analyzed in adult zebrafish acclimated to low-Ca^2+^ or high-Ca^2+^ water. After acclimation for 2d, branchial *ecac* and *vdra* mRNA expressions of adult zebrafish were significantly stimulated by low-Ca^2+^ water (Fig. 1C). On the contrary, *pmca2*, *ncx1b*, and *vdrb* mRNA expressions were not affected by environmental Ca^2+^ levels (Fig. 1C).

### Effects of Exogenous 1α,25(OH)_2_D_3_ on Ca^2+^ Influx/contents and mRNA Expressions of Ca^2+^-related Genes in Embryos

**Figure 2:**
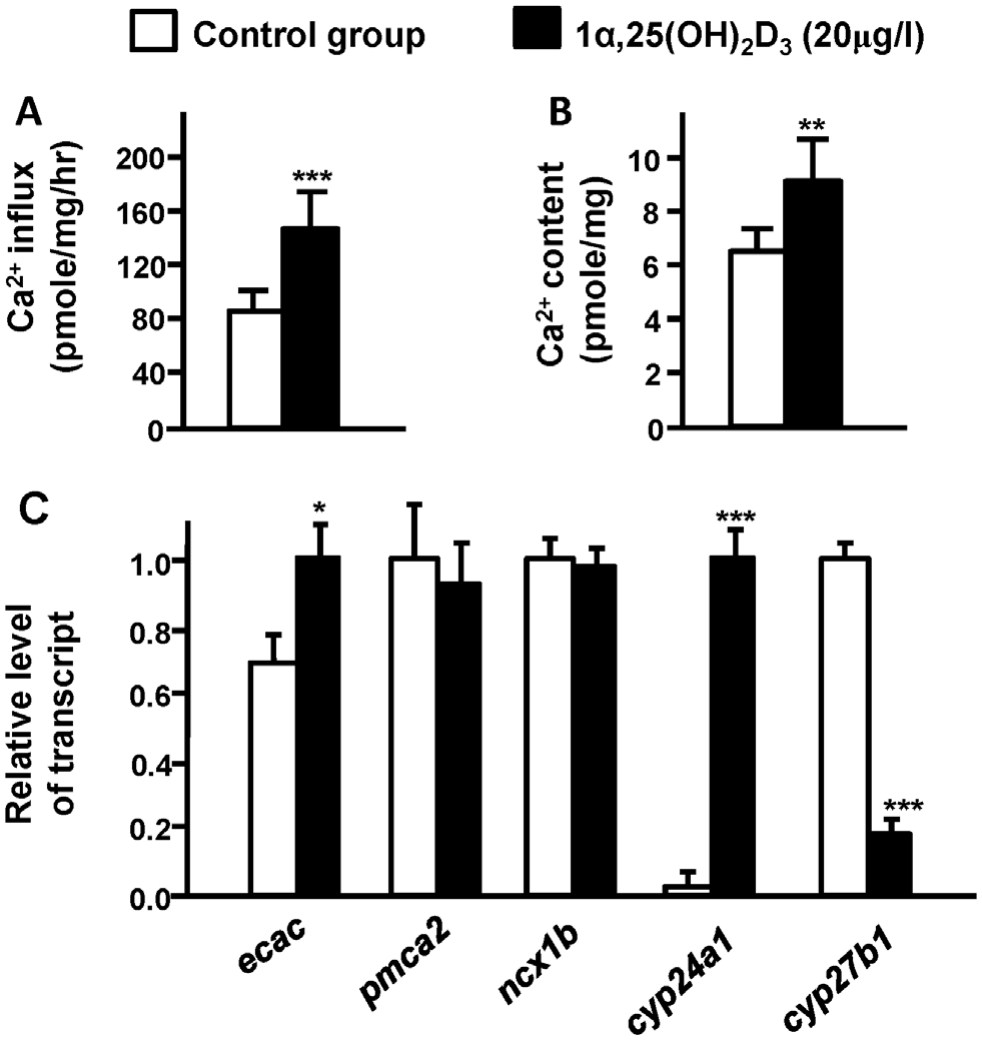
Effects of exogenous 1α,25(OH)_2_D_3_ (20 µg/l) in 3-d post-fertilization (dpf) zebrafish embryos. Ca^2+^ influx **(A)**, Ca^2+^ content (B), mRNA expressions in 3-dpf zebrafish embryos (C). mRNA expressions were analyzed by a qPCR, and values were normalized to β-actin. Values are the mean ± SD (*n* = 7∼10). Student’s *t-test*, *p<0.05; **p<0.01, ***p<0.001.

To test the hypothesis of whether vitamin D affects Ca^2+^ uptake, zebrafish embryos started to be treated with 1α,25(OH)_2_D_3_ at the 1∼2-cell stage and lasted 3 d. Compared to the control group (0 µg/l), 1α,25(OH)_2_D_3_-treated groups (20 µg/l) showed significant increases in Ca^2+^ contents and influxes in 3-dpf embryos (Fig. 2A, B). The qPCR analysis revealed differential effects of 1α,25(OH)_2_D_3_ on mRNA expressions of Ca^2+^ transporters. mRNA expressions of zebrafish *ncx1b* and *pmca2* were not affected by 1α,25(OH)_2_D_3_ treatment (Fig. 2C); however, that of *ecac* was significantly upregulated by 1α,25(OH)_2_D_3_ in 3-dpf embryos (Fig. 2C). Exogenous 1α,25(OH)_2_D_3_ also caused differential effects on mRNA expressions of *cyp24a1* and *cyp27b1* in zebrafish embryos. The qPCR analysis in 3-dpf embryos showed that exogenous 1α,25(OH)_2_D_3_ significantly inhibited the mRNA expression of *cyp27b1*, but significantly stimulated *cyp24a1*expression (Fig. 2C).

### Characterization of Zebrafish (z)VDRa and VDRb

**Figure 3:**
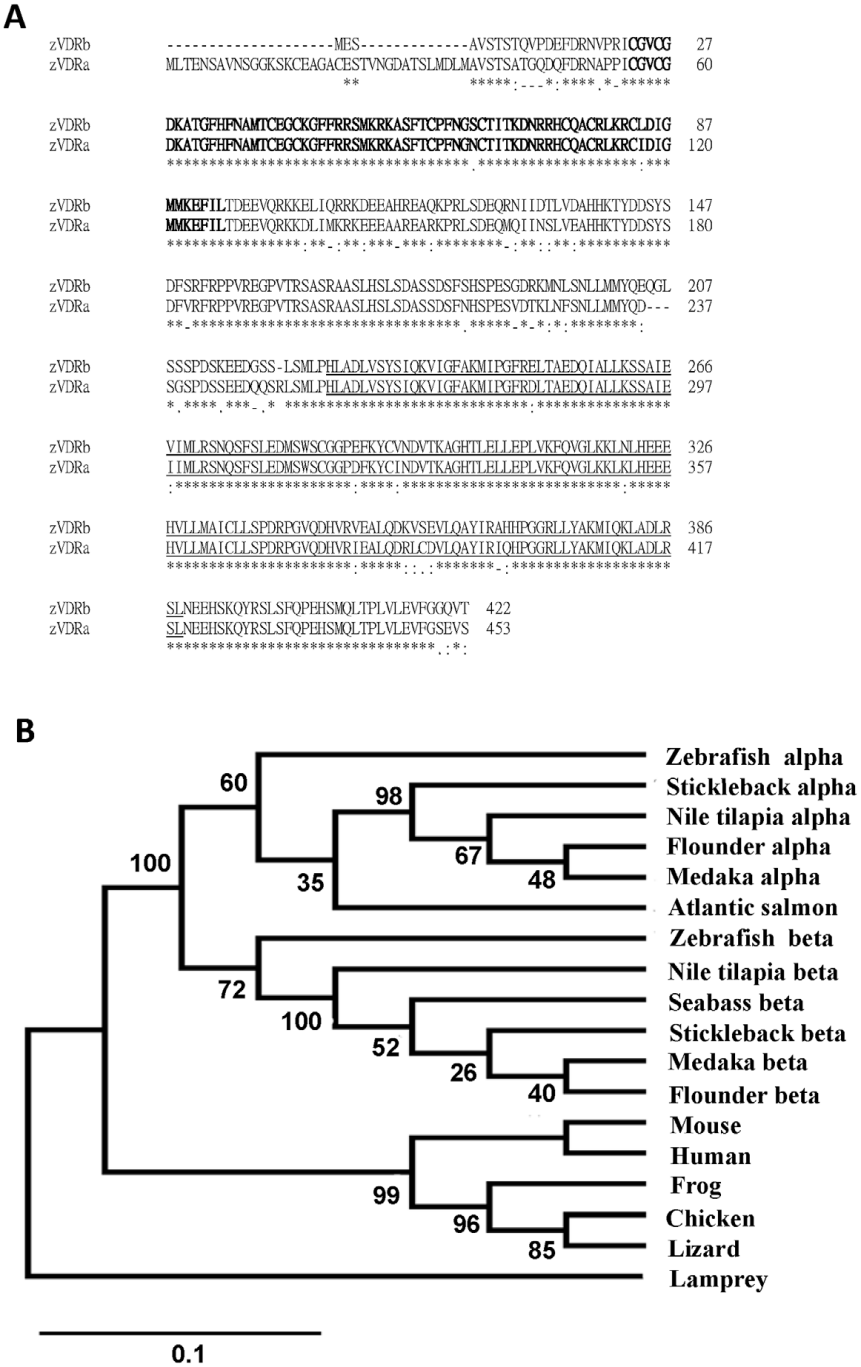
Alignment and phylogenetic analysis of vitamin D receptors (VDRs). (A) Alignment of amino-acid sequences of zebrafish (z)VDRa and VDRb. (B) Phylogenetic analysis of vertebrate VDRs. The consensus line denotes a consensus (asterisk), similarity (: or.), or difference (-) between zVDRa and zVDRb. Bold letters indicate the DNA-binding domain, and underlined letters indicate the ligand-binding domain. Phylogenetic analyses were conducted using MEGA5. The phylogenetic analyses were inferred using Neighbor-joining trees and were bootstrapped (600 pseudosamples) to assess the robustness. The percentage of replicate trees in which the associated taxa clustered together in the bootstrap test is shown next to the branches. The unit of the scale bar is the number of amino-acid substitutions per site.

zVDRa (NP_570994) and zVDRb (NP_001153457) were identified from NCBI. The 2 VDRs have 453 (zVDRa) and 422 (zVDRb) amino acids (Fig. 3A), and calculated respective molecular weights of 50.8 and 47.8 kDa. There was 86% homology in amino-acid sequences between zVDRa and zVDRb, and both zVDRa and zVDRb showed 97% homology in the DNA-binding domain (DBD; Table S2) and 92% homology in the ligand-binding domain (LBD; Table S2). zVDRa shared 88%, 85%, 89%, 85%, 68%, 70%, 65%, 71%, and 69% identities with medaka VDRα and VDRβ, flounder VDRa and VDRb, frog VDR, lizard VDR, chicken VDR, mouse VDR and human VDR, while zVDRb shared 85%, 87%, 85%, 87%, 67%, 69%, 70%, 69%, and 70% identities, respectively. A further analysis of important regions of the VDR among different species revealed that both zVDRa and zVDRb shared 93%∼97% amino-acid sequence identities with others species at the DBD, and 79%∼94% identities at the LBD (Table S2). According to the phylogenetic analysis of zVDRa and zVDRb sequences (Fig. 3B), the 2 zebrafish receptor sequences were classified into 2 clades representing teleost VDRa and VDRb, respectively. However, the 2 teleost VDR groups diverged from those of terrestrial vertebrates (Fig. 3B).

### mRNA Expressions of Vdra and Vdrb in Developing Embryos and Tissues

**Figure 4:**
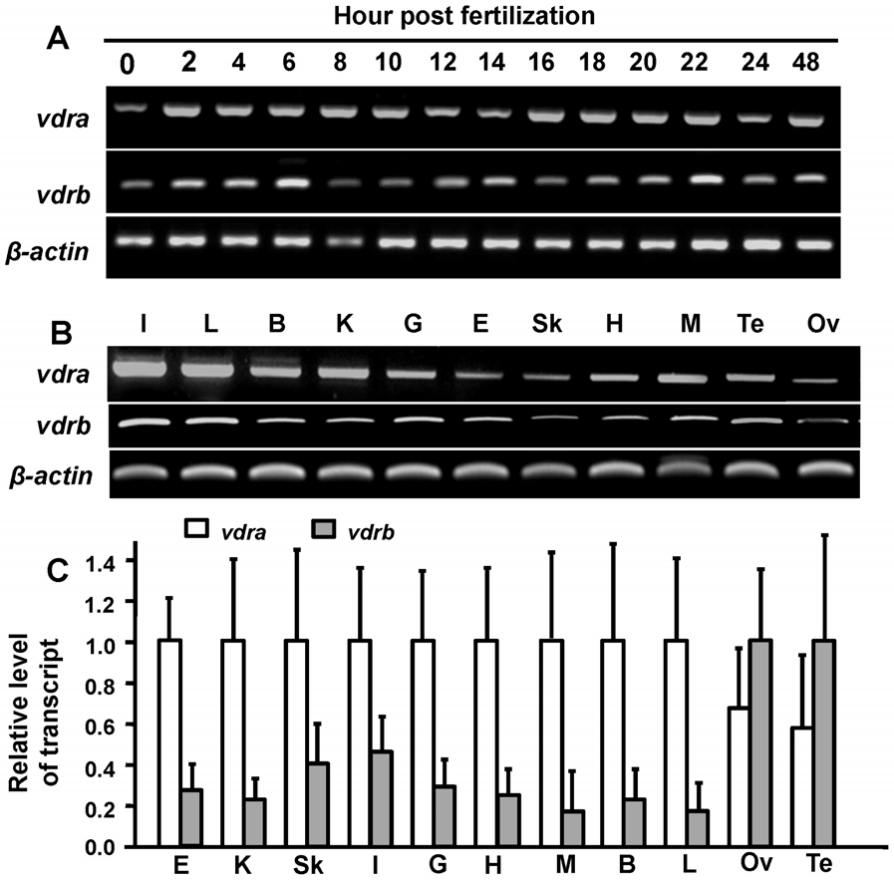
Zebrafish *vdra* and *vdrb* expression profiles. mRNA expression patterns in developing stages (A) and various tissues (B) were determined by an RT-PCR using β-actin as the internal control. (C) *vdra* and *vdrb* mRNA expressions in different tissues of adult zebrafish were analyzed by a qPCR using β-actin as the internal control. Values are the mean ± SD (*n* = 3). E, eye; K, kidney; Sk, skin; I, intestine; G, gill; H, heart; M, muscle; B, brain; L, liver; Ov, ovary; Te, testis.

Both *vdra* and *vdrb* mRNA expressions were first detected by an RT-PCR at 0 h post-fertilization (hpf) and throughout development (Fig. 4A). The RT-PCR was also used to detect *vdra* and *vdrb* mRNA expressions in different tissues. Both *vdra* and *vdrb* expressions were detected in all of the tissues examined (Fig. 4B). On the other hand, *vdra* expression was more dominant than that of *vdrb* (over 2-fold higher) in all tissues except the testes and ovaries by the qPCR analysis (Fig. 4C).

### Effects of VDRa/b Loss-of-function on Ca^2+^ Contents, Influx, and Transporters in Embryos

To block the endogenous vitamin D signaling pathway, VDRa and VDRb MOs were used to respectively inhibit translation of zebrafish VDRa and VDRb. A Western blot analysis was used to demonstrate MO specificity. As a result, VDRa and VDRb MOs were respectively found to downregulate VDRa and VDRb protein levels in 3-dpf zebrafish embryos (Fig. S1).

**Figure 5:**
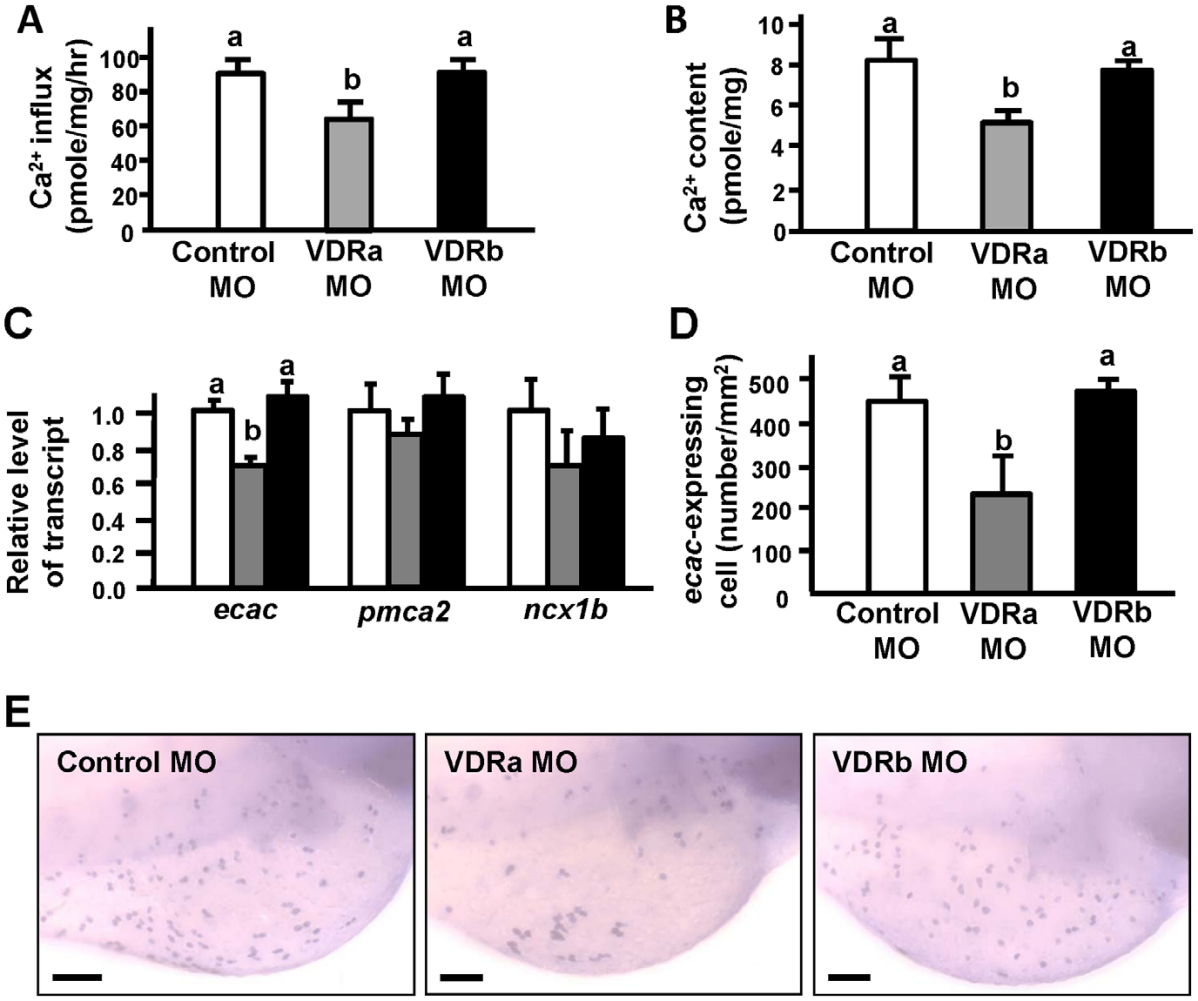
Effects of vitamin D receptor (VDR)a and VDRb morpholino oligonucleotides (MOs) in 3-d post-fertilization (dpf) zebrafish embryos. Ca^2+^ influx (A), Ca^2+^ content (B), mRNA expression (C), density of *ecac*-expressing cells (D), and *ecac* signals (E). mRNA expressions were analyzed by a qPCR using β-actin as the internal control. Different letters indicate a significant difference (*p*<0.05) using a one-way ANOVA followed by Tukey’s multiple-comparison test. Values are the mean ± SD (*n* = 6 or 7). Scale bar:100 µm.

After specificity tests, respective MOs were injected into 1∼2-cell-stage embryos. Compared to the control MO, the VDRa MO caused significant decreases in the Ca^2+^ content and influx in 3-dpf zebrafish embryos, but the VDRb MO had no effects (Fig. 5A, B). The qPCR assay of mRNA expressions of Ca^2+^ transporters showed that the VDRa MO significantly reduced the expression of *ecac*, but did not affect *ncx1b* or *pmca2* mRNA expressions in 3-dpf zebrafish embryos (Fig. 5C). In contrast with the VDRa MO, the VDRb MO did not affect expressions of *ncx1b*, *pmca2*, or *ecac* genes in 3-dpf zebrafish embryos (Fig. 5C). To further support these data, the intensity of *ecac* mRNA signals and density of *ecac*-expressing cells in the skin of zebrafish morphants were analyzed. In situ hybridization showed that the VDRa MO, but not the VDRb MO, suppressed *ecac* mRNA signals in embryonic skin. The density of *ecac*-expressing cells also only significantly decreased with a VDRa MO injection (Fig. 5D, E).

Vanoevelen et al. [2] demonstrated that an *ecac* mutant resulted in delayed bone formation in zebrafish. In the present study, VDRa/b morphants showed different effects on *ecac* expression and Ca^2+^ regulation (Fig. 5), and therefore subsequent experiments were designed to test if the VDR was involved in bone formation in zebrafish. According to results of vertebrae staining, the VDRa MO delayed ossification of vertebrae in morphants at 5 dpf, but the VDRb MO did not show a significant effect (Fig. S2), supporting the above results of different functions of the 2 VDR paralogs.

### Effects of VDRa/b Loss-of-function on Ca^2+^ Influx and Ecac mRNA Expression in Embryos Treated with 1α,25(OH)_2_D_3_ or Exposed to Low-Ca^2+^ Medium

**Figure 6:**
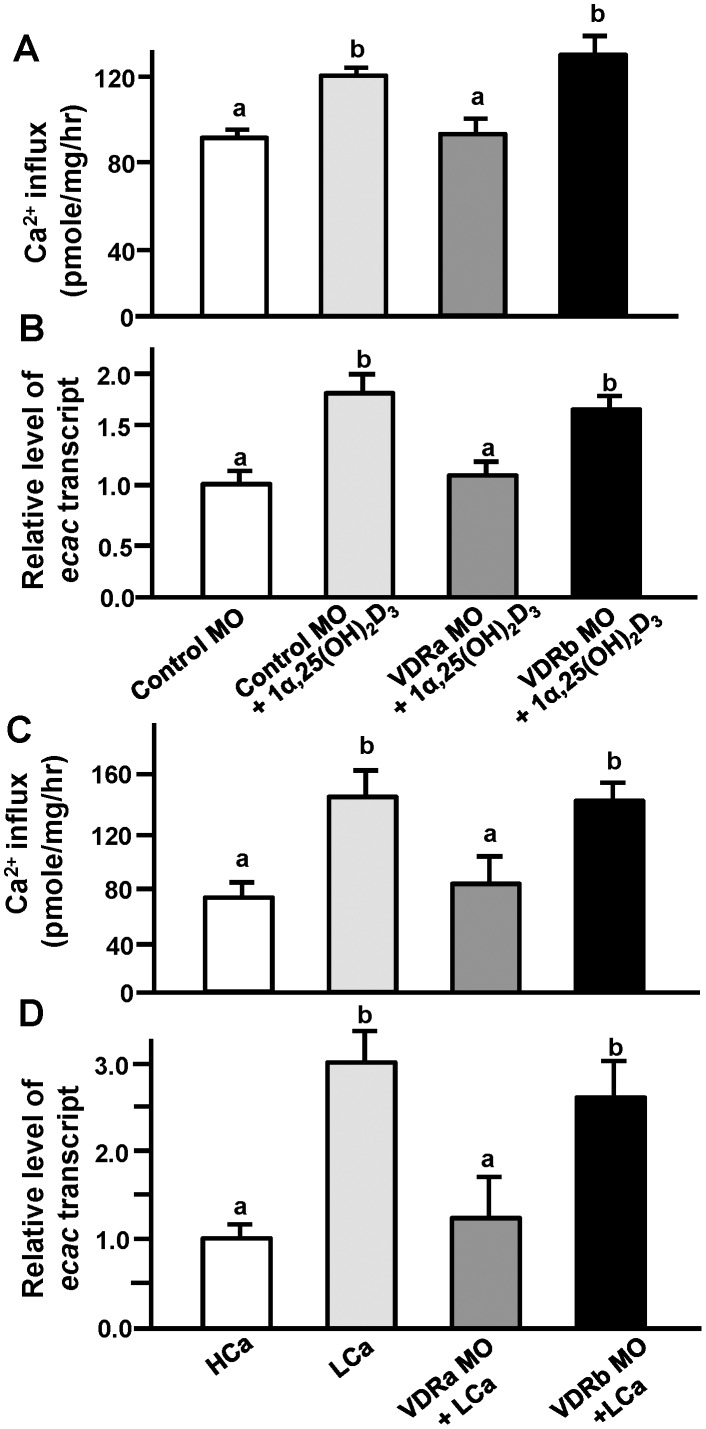
Effects of vitamin D receptor (VDR)a and VDRb morpholino oligonucleotides (MOs) on zebrafish embryos with 1α,25(OH)_2_D_3_ (20 µg/l) or low Ca^2+^ (0.02 mM; LCa) treatment. Ca^2+^ influx (A, C) and *ecac* mRNA expression (B, D). mRNA expression of *ecac* was analyzed by a qPCR using β-actin as the internal control. Different letters indicate a significant difference (*p*<0.05) using one-way ANOVA followed by Tukey’s multiple-comparison test. Values are the mean ± SD. (*n* = 6∼8). High Ca^2+^ (HCa): 2.00 mM.

To precisely ascertain the different roles of zebrafish VDRa and VDRb, zebrafish were incubated with or without 1α,25(OH)_2_D_3_ (20 µg/l) after injecting the MOs. Compared to the control group (control MO injection without 1α,25(OH)_2_D_3_), both groups of the control MO with 1α,25(OH)_2_D_3_ and the VDRb MO with 1α,25(OH)_2_D_3_ exhibited significantly higher Ca^2+^ influxes at 3 dpf, but VDRa MO-injected embryos with 1α,25(OH)_2_D_3_ showed no difference (Fig. 6A). Similarly, *ecac* mRNA expression in the control MO with 1α,25(OH)_2_D_3_ and the VDRb MO with 1α,25(OH)_2_D_3_ was significantly stimulated, while that of the VDRa MO-injected embryos was not affected by 1α,25(OH)_2_D_3_ (Fig. 6B).

Low-Ca^2+^ medium is known to stimulate Ca^2+^ influx and *ecac* expression in zebrafish [32], [33] (Fig. 6C, D).Whether this Ca^2+^ influx and *ecac* expression were upregulated by low-Ca^2+^ medium as mediated by VDRa or VDRb was further clarified in the following experiments. Embryos at the 1∼2-cell stage were respectively injected with the control MO, VDRa MO, and VDRb MO, and then incubated in 2.0 mM (high) or 0.02 mM (low) Ca^2+^ medium. Compared to the control MO in low-Ca^2+^ medium, VDRa morphants in low-Ca^2+^ medium had significantly lower Ca^2+^ influx and *ecac* mRNA expression at 3 dpf, but VDRb morphants in low-Ca^2+^ medium had similar values to the control group (Fig. 6C, D).

### Colocalization of VDRa with Na,K-ATPase-rich (NaR) Cells

There are at least three subtypes of ionocytes, NaR (Na^+^,K^+^-ATPase-rich) cells, HR (H^+^-ATPase-rich) cells, and NCC (Na^+^/Cl^−^ cotransporter expressing) cells in zebrafish gill/skin ionocytes [4]. NaR cells, which expresses ECaC, PMCA2, and NCX1b, is mainly responsible for the Ca^2+^ uptake function in the skin of developing embryos and gills of adults [4]. To reinforce the above molecular physiological evidence for the involvement of vitamin D-VDRa signaling in zebrafish Ca^2+^ uptake function, we further tested the hypothesis of whether VDRa is expressed in NaR ionocytes by double in situ hybridization/immunocytochemistry for *vdra* mRNA and Na,K-ATPase (a marker of NaR cells) in zebrafish gills. As shown in Fig. S3, most of the *vdra* mRNA signals were colocalized in NaR ionocytes that were labeled with Na,K-ATPase.

### Effects of VDRa/b Loss-of-function on mRNA Expressions of cyp27b1 and cyp24a1 in Embryos Treated with 1α,25(OH)_2_D_3_

**Figure 7:**
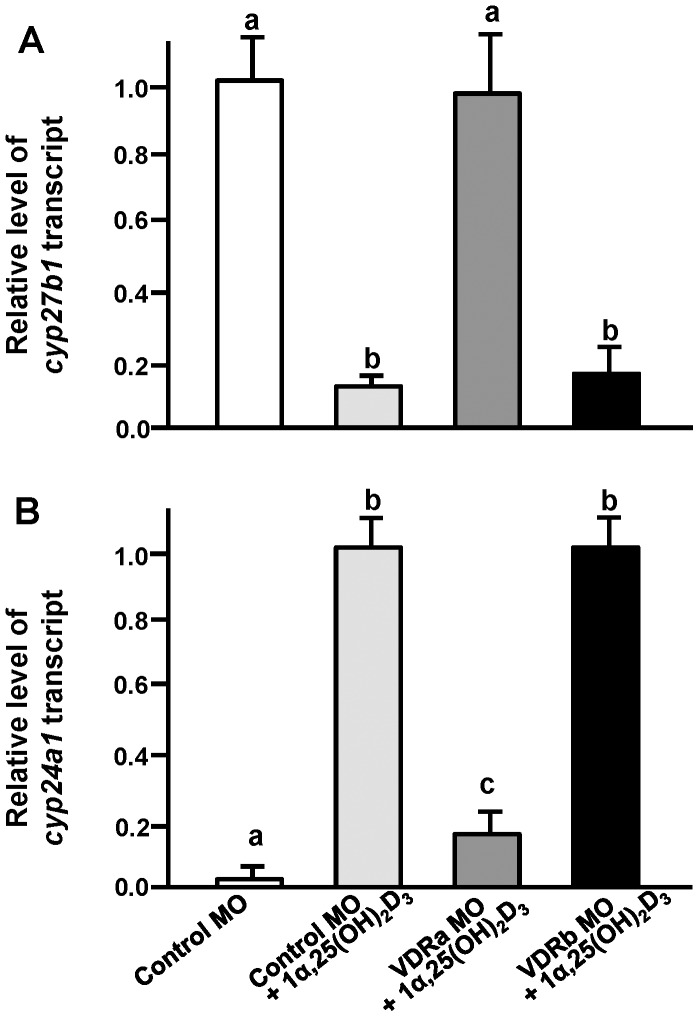
Effects of vitamin D receptor (VDR)a and VDRb morpholino oligonucleotides (MOs) on *cyp27b1* and *cyp24a1* mRNA expressions in 3-d post-fertilization (dpf) zebrafish embryos treated with 1α,25(OH)_2_D_3_ (20 µg/l). (A) *cyp27b1* mRNA expression. (B) *cyp24a1* mRNA expression. mRNA expressions were analyzed by a qPCR using β-actin as the internal control. Different letters indicate a significant difference (*p*<0.05) using one-way ANOVA followed by Tukey’s multiple-comparison test. Values are the mean ± SD. (*n* = 5–6).

The above experiments (Fig. 2) showed different effects of exogenous 1α,25(OH)_2_D_3_ on expressions of *cyp27b1* and *cyp24a1* in zebrafish embryos. A subsequent experiment was designed to further clarify if 1α,25(OH)_2_D_3_ regulates expressions of *cyp27b1* and *cyp24a1* through mediation by the VDR in zebrafish as in mammals [35]–[37]. Embryos at the 1∼2-cell stage were injected with the control MO, VDRa MO, and VDRb MO, respectively, and then incubated without or with 20 µg/l 1α,25(OH)_2_D_3_. The VDRb MO could not neutralize effects of exogenous 1α,25(OH)_2_D_3_ on mRNA expressions of *cyp27b1* and *cyp24a1* in 3-dpf zebrafish embryos (Fig. 7A, B). On the contrary, the VDRa MO modulated *cyp27b1* and *cyp24a1* expressions (Fig. 7A, B). The VDRa MO neutralized both 1α,25(OH)_2_D_3_-downregulated *cyp27b1* mRNA expression (Fig. 7A) and 1α,25(OH)_2_D_3_-upregulated *cyp24a1* mRNA expression in zebrafish embryos (Fig. 7B).

## Discussion

Besides the source from food and drinking, teleostean fish actively absorb Ca^2+^ from the aquatic environment with fluctuating in Ca^2+^ levels [5]. Therefore, body fluids Ca^2+^ homeostasis and bone formation (particularly in embryonic and larval stages) in freshwater teleosts must be strictly regulated to cope with a fluctuating environment. Vitamin D increased plasma calcium levels in cod [19] and caused dose-dependent hypercalcemia in carp [18]. Lock et al. (2007) suggested a crucial role of the vitamin D system in Ca^2+^ handling in Atlantic salmon because 1α,25(OH)_2_D_3_ concentrations and VDR mRNA expressions changed in salmon undergoing smoltification and migrating from fresh water (low calcium concentrations) to seawater (high calcium concentrations) [21]. Like other teleosts, zebrafish can enhance their Ca^2+^ uptake function by stimulating ECaC expression during acclimation to low-Ca^2+^ fresh water [32]–[34], and this functional regulation was further demonstrated to be mediated by vitamin D-VDR signaling in the present study. Low-Ca^2+^ fresh water stimulated mRNA expressions of *ecac*, *vdra*, *vdrb,* and *vitamin D-25hydroxylase* (*cyp2r1*) in 3- and/or 5-dpf zebrafish embryos, implying a possible role of vitamin D in the functional control of Ca^2+^ uptake in zebrafish. To test this hypothesis, we treated zebrafish with exogenous 1α,25(OH)_2_D_3_, which was found to stimulate the mRNA expression of *ecac* and Ca^2+^ influx and result in increased total calcium contents in the whole body. The following experiments from the molecular to the physiological level demonstrated the calciotropic effects of vitamin D in zebrafish.

Qiu et al., (2007) showed that 1α,25(OH)_2_D_3_ stimulated branchial *ecac* mRNA in trout, but they did not attempt to examine other Ca^2+^ transporters (NCX or PMCA) [38]. In zebrafish, a specific type of ionocyte that expresses ECaC, PMCA2, and NCX1b were identified to be responsible for the transepithelial Ca^2+^ uptake function [3], [4], [8], [32], [33], providing a suitable model to identify the exact target transporter(s) of vitamin D in Ca^2+^ uptake mechanisms. The present study first reports that 1α,25 (OH)_2_D_3_ only regulates the expression of *ecac* but not that of *ncx1b* or *pmca2*. These results support a previous notion that ECaC is the major regulatory player in the epithelial Ca^2+^ uptake pathway in fish [4] as in mammals [39]. Similarly, both hypercalcemic cortisol and hypocalcemic stanniocalcin 1(STC1) were also found to control Ca^2+^ influx by regulating the expression of *ecac,* but neither affected that of *ncx1b* or *pmca2* in zebrafish [17], [29]. *ecac* appears to be the major regulatory target transporter gene not only in response to environmental Ca^2+^ but also in control pathways of related hormones in zebrafish.

Most physiological functions of vitamin D signaling are mediated by the VDR, which is a ligand-activated transcription factor [35]. Because of genome duplication, teleosts have 2 paralogous VDR forms [24], [25]. Craig et al. (2008) immunohistochemically demonstrated universal expression of the zebrafish VDR in most tissues; however, they did not try to identify the respective expressions of paralogous VDRs [40]. In the present study, 2 paralogous VDRs with a high degree of homology (86%) were also identified in zebrafish. In the phylogenetic analysis, the 2 paralogous VDRs of zebrafish were separated into 2 clades. The 2 paralogous VDRs were found to be expressed throughout the developmental stages and adult tissues of zebrafish, and VDRa showed a predominant expression over VDRb in most tissues. Although most gene duplicates are non-functionalized or the gene is eventually lost, some paralogous genes are preserved as one acquires new function or subfunction [26]. There has been no convincing evidence to answer the long-term challenging question: do the 2 paralogous VDRs in teleosts have divergent functions? In previous studies, exogenous 1α,25(OH)_2_D_3_ treatment was found to stimulate transcription of VDRE-containing expression constructs in a mammalian cell line that overexpressed the teleost VDR [24], [27], [28]. Howarth et al (2008) further demonstrated in vitro that transcriptional regulation of 2 paralogous VDRs of medaka differed with 1α,25(OH)_2_D_3_ treatment [24]. Those studies indicated that vitamin D can differentially activate teleost VDRs; however, it was unknown until the present study that vitamin D controls the Ca^2+^-uptake function through only one of the paralogous receptor genes, VDRa, in zebrafish based on the loss-of-function experiments of the 2 VDRs.

The teleost VDR simulated the transcript level of the VDRE-containing construct with 1,25(OH)_2_D_3_ treatment in a cell line experiment [24], [27], [28]. Furthermore, a putative VDRE was identified in the *ecac* promoter region of fugu and zebrafish [17], [23]. Taken together, vitamin D-VDRa signaling is probably involved in controlling the expression and function of *ecac* in fish. In the present study, paralogous VDRs of zebrafish were differentially activated by vitamin D in the control pathways of Ca^2+^ uptake and *ecac* expression, implying some divergences in the functions between these two paralogous VDRs in zebrafish, as what was previously reported using *in vitro* experiments on the 2 medaka VDRs [24]. To ascertain functional information on the paralogous VDRs, Horwarth et al. (2008) constructed chimeric proteins containing the yeast Gal4 DNA-binding domain (DBD) fused with the VDR ligand-binding domain (LBD) of either medaka VDRα or VDRβ [24]. Activity of the medaka VDRα chimera exhibited little activation by 1,25(OH)_2_D_3_, but a stronger and more-specific response was observed in the VDRβ chimera [24]. In the present study, paralogous VDRs of zebrafish showed different activation extents by 1,25(OH)_2_D_3_, and this may have resulted from the difference of amino-acid sequence in the LBD. Comparison of amino-acid compositions between zebrafish VDRa and VDRb showed a higher degree of similarity in the DBD (∼97%) than LBD (∼92%), demonstrating that slight changes in the amino-acid composition may be associated with a significant difference in transactivation and thus physiological functions.

Vitamin D was found to directly stimulate the *cyp24a1* transcript and inhibit the *cyp27b1* transcript through its binding to the VDR in experiments on mammalian cell lines [24]. However, this regulation is still unclear in teleosts. In the present study, exogenous vitamin D (1α,25(OH)_2_D_3_) suppressed the mRNA expression of *cyp27b1* and simultaneously stimulated that of *cyp24a1* in 3-dpf zebrafish embryos, reflecting a feedback mechanism in homeostasis of vitamin D levels as found in mammals. In experiments on mammals or mammalian cell lines, exogenous 1α,25(OH)_2_D_3_ caused negative feedback which directly suppressed gene expression and activity of CYP27B1 [41]–[43]. On the other hand, exogenous 1α,25(OH)_2_D_3_ caused positive feedback to directly stimulate gene expression of CYP24A1 [43], [44]. This regulation can be associated with the homeostasis of 1α,25(OH)_2_D_3_. Accordingly, stimulation of *cyp24a1* and inhibition of *cyp27b1* by exogenous 1,25(OH)_2_D_3_ treatment may provide feedback to control 1α,25(OH)_2_D_3_ levels in zebrafish. Taken together, 1α,25(OH)_2_D_3_ may regulate the function of the Ca^2+^ uptake mechanism in fish through feedback pathways, in which expressions of *cyp27b1* and *cyp24a1* are differentially modulated. Subsequent loss-of-function experiments further indicated that this feedback control by the differential regulation of *cyp27b1* and *cyp24a1* expressions appears to be mediated by only one of the paralogous receptors, VDRa, supporting results of previous experiments (see the preceding paragraph). Loss-of-function of VDRa was found to modulate exogenous 1α,25(OH)_2_D_3_-induced changes in *cyp24a1* and *cyp27b1*expressions in zebrafish, while that was not the case in VDRb morphants. Notably, a continuous feedback effect of 1α,25(OH)_2_D_3_ appeared to cause an inhibitory effect on the Ca^2+^ uptake capacity through downregulation of *ecac* expression in 5-dpf embryos to prevent excess Ca^2+^ uptake (unpublished data).

In summary, vitamin D-VDRa signaling was demonstrated to stimulate Ca^2+^ uptake by upregulating ECaC in zebrafish through feedback pathway associated with the differential regulation of CYP27B1and CYP24A1. Compared to VDRa, VDRb seems to be nonfunctional in calcemic regulation of zebrafish. The present study for the first time clarifies the divergent physiological functions of paralogous VDRs in a teleost after a gene-duplication event. Similar actions of vitamin D on Ca^2+^ homeostasis evolved in zebrafish as in mammals, and thus zebrafish may serve as a model to explore the function of vitamin D-VDR signaling in Ca^2+^ homeostasis and related physiological processes in vertebrates.

## Supporting Information

Figure S1 **Specificity and effectiveness of vitamin D receptor (VDR)a and VDRb morpholino oligonucleotides (MOs).** VDRa and VDRb MOs were respectively injected into 1- or 2-cell embryos. To clarify the MO specificity and effectiveness, Western blotting was used to detect VDRa and VDRb protein expressions in wild-type (WT) and MO-injected embryos at 3 d post-fertilization (dpf). An asterisk (*) indicates the position of VDRa or VDRb expression.
(TIF)

Figure S2 **Effect of vitamin D receptor (VDR)a and VDRb morpholino oligonucleotides (MOs) on ossification of vertebrae of 5-d post-fertilization (dpf) zebrafish embryos.** Control (A), VDRa (B), and VDRb (C) MO. Ossification of vertebrae was observed by calcein staining. Scale bar: 100 µm.
(TIF)

Figure S3 **Colocalization of** ***vdra*** **mRNA with Na,K-ATPase rich (NaR) cells in zebrafish gill cryosections.** (A) In situ hybridization of *vdra* mRNA; (B) immunocytochemical staining of Na,K-ATPase. The arrow indicates colocalization of *vdra* mRNA and Na,K-ATPase protein signals in the same cells. Scale bar: 5 µm.
(TIF)

Table S1 **Primers for the RT-PCR and qPCR analyses.**
(DOCX)

Table S2 **Identities (%) of amino-acid sequences among the DNA-binding domain (DBD) and ligand-binding domain (LBD) of vitamin D receptors (VDRs) in different species.**
(DOCX)
