# Supplementary figures and images for: Action of Vitamin D and the Receptor, VDRa, in Calcium Handling in Zebrafish (Danio rerio)
Source: PLoS One. 2012 Sep 19;7(9):e45650. doi: 10.1371/journal.pone.0045650 (PMC3446910; doi:10.1371/journal.pone.0045650)

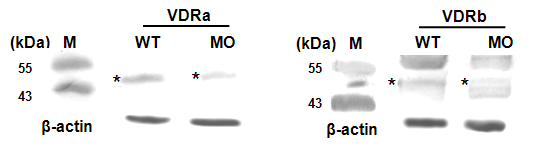

Supplement: Figure S1 — Specificity and effectiveness of vitamin D receptor (VDR)a and VDRb morpholino oligonucleotides (MOs). VDRa and VDRb MOs were respectively injected into 1- or 2-cell embryos. To clarify the MO specificity and effectiveness, Western blotting was used to detect VDRa and VDRb protein expressions in wild-type (WT) and MO-injected embryos at 3 d post-fertilization (dpf). An asterisk (*) indicates the position of VDRa or VDRb expression. (TIF) [file pone.0045650.s001.tif]

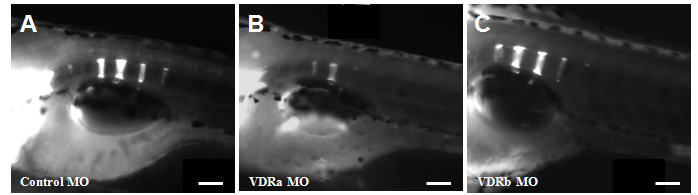

Supplement: Figure S2 — Effect of vitamin D receptor (VDR)a and VDRb morpholino oligonucleotides (MOs) on ossification of vertebrae of 5-d post-fertilization (dpf) zebrafish embryos. Control (A), VDRa (B), and VDRb (C) MO. Ossification of vertebrae was observed by calcein staining. Scale bar: 100 µm. (TIF) [file pone.0045650.s002.tif]

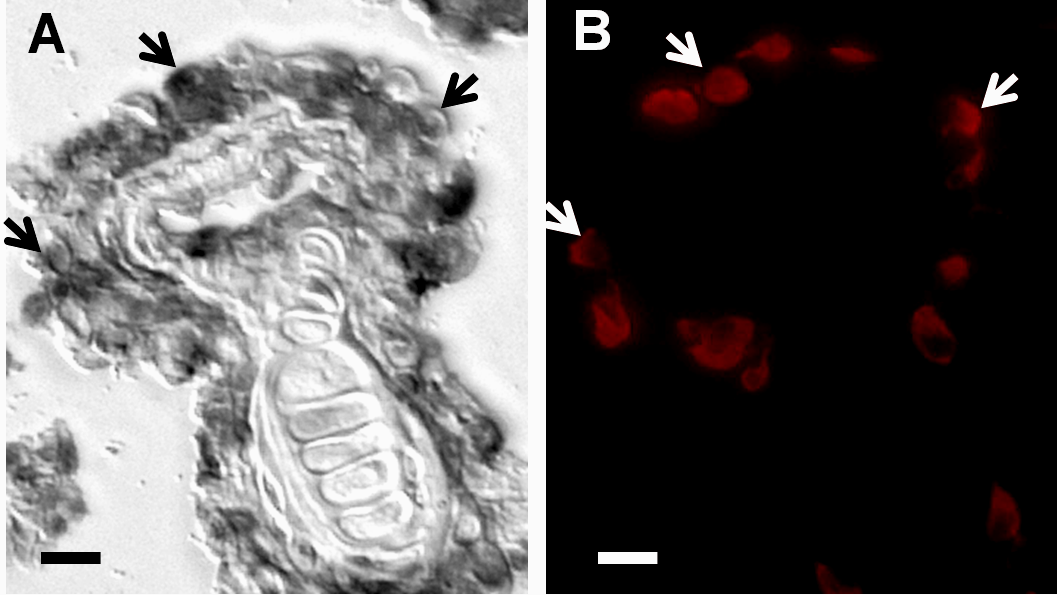

Supplement: Figure S3 — Colocalization of vdra mRNA with Na,K-ATPase rich (NaR) cells in zebrafish gill cryosections. (A) In situ hybridization of vdra mRNA; (B) immunocytochemical staining of Na,K-ATPase. The arrow indicates colocalization of vdra mRNA and Na,K-ATPase protein signals in the same cells. Scale bar: 5 µm. (TIF) [file pone.0045650.s003.tif]
