# Supplementary material for: Action of Vitamin D and the Receptor, VDRa, in Calcium Handling in Zebrafish (Danio rerio)
Source: PLoS One. 2012 Sep 19;7(9):e45650. doi: 10.1371/journal.pone.0045650 (PMC3446910; doi:10.1371/journal.pone.0045650)
Supplement: Table S1 — Primers for the RT-PCR and qPCR analyses. (DOCX) [file pone.0045650.s004.docx]

**Table S1. Primers for the RT-PCR and qPCR analysis**

| Gene name |  | Primer sequence | |
| --- | --- | --- | --- |
| *ecac* | F | 5' TCCTTTCCCATCACCCTCT 3' | |
|  | R | 5' GCACTGTGGCAACTTTCGT 3' | |
| *pmca2* | F | 5' AAGCAGTTCAGGGGTTTAC 3' | |
|  | R | 5' CAGATCATTGCCTTGTATCA3' | |
| *ncx1b* | F | 5' TAAAGTGGCAGCGATACAGGT 3' | |
|  | R | 5' CAGATCAAGGCGAAGATGG3' | |
| *vdra* | F | 5' CTCGGATTCTGTGGATGCTT 3' | |
|  | R | 5' GGCCTTACGCTTCATACTGC 3' | |
| *vdrb* | F | 5' ACACAGCGTGGAGTGGAGT 3' | |
|  | R | 5' ACACTCCATGGCAAGAACA 3' | |
| *cyp2r1* | F | 5' TTGAAGACAGGCAGAGGATG 3' | |
|  | R | 5' GGGTCGCTCCAGTACTTCTC 3' | |
| *cyp27b1* | F | 5' TCTATCCTGTTATTCCAGCCAA 3' | |
|  | R | 5' GCCTGAAGGAGTCTGGATCT 3' | |
| *cyp24a1*  *b-actin* | F  R  F | 5' AAAGAGGGCAGCTATCCTGA 3'  5' CATGAGCTTCTGCTGGAAAG 3'  5' ATTGCTGACAGGATGCAGAAG 3' | |
|  | R | 5' GATGGTCCAGACTCATCGTACTC 3' | |
|  |  |  |  |

The accession numbers of nucleic acid sequences are as followed: *ecac* [GenBank:NM_001001849], *pmca2* [GenBank:NM_001123238], *ncx1b* [GenBank:NM_001039144], *vdra* [GenBank:NM_130919], *vdrb* [GenBank:NM_001159985], *cyp2r1* [GenBank:XM_686732], *cyp27b1* [EMBL:ENSDART00000066178], *cyp24a1* [GenBank:NM_001089458], *β-actin* [GenBank:NM_181601]. F:Fordward, R:Reverse.
