# Supplementary material for: Action of Vitamin D and the Receptor, VDRa, in Calcium Handling in Zebrafish (Danio rerio)
Source: PLoS One. 2012 Sep 19;7(9):e45650. doi: 10.1371/journal.pone.0045650 (PMC3446910; doi:10.1371/journal.pone.0045650)
Supplement: Table S2 — Identities (%) of amino-acid sequences among the DNA-binding domain (DBD) and ligand-binding domain (LBD) of vitamin D receptors (VDRs) in different species. (DOCX) [file pone.0045650.s005.docx]

**Table S2. Identities (%) of amino acid sequence among the DNA binding domain (DBD) and ligand binding domain (LBD) of vitamin D receptor (VDR) in different species**

**DBD LBD**

**zVDRa**   **zVDRb zVDRa zVDRb**

**zVDRa**  -- 97 -- 92

**zVDRb --** 97 -- -- 92 --

**mVDRα**  97 97 94 93

**mVDRβ**  97 98 89 90

**fVDRa**  98 98 94 92

**fVDRb**  94 95 90 90

**frVDR** - 93 93 80 80

**lVDR** 94 94 81 - 81

**cVDR**  93 93 83 83

**mVDR** 93 93 79 - 79

**hVDR-**  93 93 80 79

**z: zebrafish, m: medaka, f: flounder, fr: frog, l: lizard, c: chicken, mo: mouse, h: human.**
